# Chronic Stress Produces Persistent Increases in Plasma Corticosterone, Reductions in Brain and Cardiac Nitric Oxide Production, and Delayed Alterations in Endothelial Function in Young Prehypertensive Rats

**DOI:** 10.3389/fphys.2018.01179

**Published:** 2018-08-29

**Authors:** Iveta Bernatova, Angelika Puzserova, Peter Balis, Natalia Sestakova, Martina Horvathova, Zuzana Kralovicova, Ingrid Zitnanova

**Affiliations:** ^1^Institute of Normal and Pathological Physiology, Centre of Experimental Medicine, Slovak Academy of Sciences, Bratislava, Slovakia; ^2^Institute of Medical Chemistry, Biochemistry and Clinical Biochemistry, Faculty of Medicine, Comenius University, Bratislava, Slovakia

**Keywords:** crowding stress, borderline hypertension, oxidative stress, corticosterone, nitric oxide, endothelial dysfunction

## Abstract

This study was designed to investigate whether oxidative stress, nitric oxide (NO) deficiency and/or endothelial dysfunction (ED) are present in young borderline hypertensive rats (BHR) and whether these pathologies can be causally involved in the initiation of blood pressure (BP) increases. Additionally, we tested the hypothesis that crowding stress, experienced during the peripubertal period, may produce persistent or delayed disorders in corticosterone release, NO synthesis, oxidative status and/or endothelial function that could accelerate BP increases. To test these hypotheses, 5-week-old male BHR and normotensive Wistar-Kyoto rats (WKY) were either kept in control conditions (for 2 and 4 weeks, respectively) or exposed to social stress produced by crowding for 2 weeks (stress). After cessation of crowding, a group of rats of each phenotype was kept in control conditions for the next 2 weeks (post-stress). Systolic BP of 5-week-old BHR was significantly increased vs. age-matched WKY (127 ± 3 vs. 104 ± 3 mmHg, *p* < 0.01) and remained significantly higher throughout the course of the experiment. Despite elevated BP, no signs of oxidative damage to plasma lipids, NO deficiency or ED were observed in control BHR vs. age-matched WKY. Crowding stress elevated plasma corticosterone and accelerated BP increases only in BHR; these effects persisted 2 weeks post-stress. Crowding failed to induce oxidative damage to plasma lipids in either phenotype, but it produced persistent decreases in NO production in the hypothalamus and brainstem of both strains of rats, as well as in the hearts of BHR. In contrast, crowding failed to reduce NO production in the aortae or acetylcholine-induced relaxations of the femoral arteries in both strains investigated. However, significantly reduced aortic NO production was observed in BHR 2 weeks post-stress vs. age-matched controls, which was in agreement with reduced NO-dependent components of vasorelaxation. In conclusion, this study’s data showed that oxidative stress, NO deficiency and ED are not causally involved in initiation of blood pressure increase in BHR. However, exposure to stressful environments produced persistent increases in plasma corticosterone and reductions of brain and cardiac NO production followed by a delayed decrease in the NO-dependent component of endothelium-dependent relaxation—changes that collectively accelerated BP increases only in BHR.

## Introduction

Hypertension is a widespread disease that increases in prevalence with age (Sun, 2015). However, hypertension or prehypertension (i.e., early stage of hypertension development) are also common among children and adolescents. Prehypertension prevalences of 10–15% were observed among 11- to 17-year-old adolescents (McNiece et al., 2007; Regecova et al., 2011). Even worse, in a study of 4- to 6-year-old children in Spain, the estimates of prehypertension and hypertension prevalence were 12.3 and 18.2%, respectively (Martin-Espinosa et al., 2017). Despite intensive clinical and experimental research, the causes of hypertension remain unknown in approximately 95% of all human cases. Endothelial dysfunction (ED) is a common pathology observed in hypertensive humans (Mordi et al., 2016) and rats (Bernatova, 2014). Oxidative stress and nitric oxide (NO) deficiency frequently cause ED in humans and experimental models of hypertension. Nevertheless, the roles of ED, oxidative stress and NO deficiency in the initiation of hypertension remains unclear (Bernatova et al., 2009; Bernatova, 2014).

Despite persistent discrepancies, chronic stress is widely considered to be an etiological factor associated with the development of cardiovascular diseases (Golbidi et al., 2015), especially when present in early life. Indeed, stressful events in early life increase the risk of elevated blood pressure (BP) in late adulthood (Alastalo et al., 2013). However, there is relatively little information regarding the delayed influence of stress on cardiovascular functioning in young subjects with a family history of hypertension who are genetically predisposed to BP elevation.

Spontaneously hypertensive rats (SHR) are a commonly used rodent model of human primary hypertension (Kunes et al., 2012). Since hypertension development in SHR occurs early in life, it is difficult to investigate metabolic and cardiovascular differences in the pre-hypertensive period. For this purpose, borderline hypertensive rats (BHR) are a more appropriate experimental model of human prehypertension. BHR are produced by mating SHR dams with Wistar-Kyoto (WKY) sires (Fuchs et al., 1998; Slezak et al., 2014). Studies with BHR have shown that this strain is more sensitive to stress than normotensive rats, suggesting a role of stress in the development of high BP in genetically predisposed subjects.

Various rodent experimental models of stress were developed to simulate the effect of stress on BP in order to investigate the mechanisms underlying the development of hypertension (Bouzinova et al., 2014; Crestani, 2016). In our study, we used a model of social stress produced by the crowding that results from reduced living space (Slezak et al., 2014). This model seems to be more relevant to the human condition (D’Atri et al., 1981; Fleming et al., 1987) than other experimental models of chronic stress, as it “typically evokes social stress reactions with prominent psychosocial components, mimicking emotional state alterations” (Bugajski, 1999). Although crowding is a relatively mild stressor, chronic exposure may lead to neuroendocrine, behavioral and cardiovascular alterations (Bugajski et al., 2006; Moiseeva et al., 2009; Bernatova et al., 2010).

This study was designed to investigate whether oxidative stress, NO deficiency and/or ED are present in young BHR males and whether they may be causatively involved in the initiation of BP increases. We also tested the hypothesis that sub-chronic crowding stress, experienced during the peripubertal period, can produce vascular alterations and thus accelerate BP increases in male BHR. Furthermore, we tested the hypothesis that crowding stress may produce persistent or delayed disorders in the hypothalamic-pituitary-adrenocortical (HPA) axis, NO synthesis and/or oxidative status that would produce vascular alterations and/or BP increases.

## Materials and Methods

### Animals and Stress Model

All rats used in our study were born in our animal facility in order to ensure identical conditions for all subjects. BHR were the first filial generation of offspring of SHR dams and WKY sires. Five weeks old male rats were separated from their mothers and randomly allocated into control and crowding stress-exposed groups. Control rats were kept under standard conditions [*∼*200 cm^2^/100 g of body weight (BW)] in groups of four rats per cage for two (C7) and 4 weeks (C9), respectively. Rats exposed to stress were kept in groups of five rats per cage with living space approximately 70 cm^2^/100 g of BW for 2 weeks, as described previously (Slezak et al., 2014). Five-week-old WKY (*n* = 36) and BHR (*n* = 36) were housed either in control (*n* = 16, for each phenotype) or crowded conditions (*n* = 20, for each phenotype). After crowding, rats were either killed (*n* = 10, for each phenotype, stress group) or placed back into control conditions (*n* = 10, for each phenotype) for the next 2 weeks (post-stress groups).

All rats were maintained at a temperature 22–24°C and artificial lights (12 h light/dark cycle). The animals were fed a standard pellet diet and tap water *ad libitum*. At the end of the experiment (between 08.00 and 09.00), the rats were decapitated after brief (60–90 s) CO_2_ anesthesia.

All experiments were approved by the State Veterinary and Food Administration of the Slovak Republic.

### Heart Rate and Blood Pressure

Heart rate (HR) and systolic BP were determined by tail-cuff method between 08.00 and 11.00, as described in detail previously (Puzserova et al., 2013b). Each BP and HR value was calculated as the average of five measurements. BP and HR values were measured at the beginning of the experiment (basal/day 0), and after each week of the experiment (days 7, 14, 21, and 28). BW was determined on the same days. Relative wet mass of the left heart ventricle (LHV) and both adrenal glands (AG) were determined at the end of experiment as the LHV/BW and AG/BW ratio, respectively.

### Plasma Corticosterone

Rats were killed by decapitation as described above. Trunk blood samples were collected in heparinized test tubes and immediately centrifuged at 850 g for 10 min at 4°C. Plasma samples were then stored at -80°C until analysis. Plasma corticosterone (pCort) was measured in duplicate in 20 μl of plasma by an enzyme immunoassay kit (Arbor Assays, Ann Arbor, MI, United States), as described in detail previously (Puzserova et al., 2013b).

### Nitric Oxide Synthase Activity

Activity of NO synthase (NOS) was determined by conversion of [^3^H]-L-arginine (MP Biomedicals, Santa Ana, CA, United States) in 20% crude tissue homogenates of the LHV, aorta, hypothalamus and brainstem, as described previously (Puzserova et al., 2013b), and was expressed as pmol/min/mg of protein.

### Determination of Oxidative Status

Blood was collected into heparin-coated tubes and centrifuged (4°C, 850 g for 10 min) to obtain erythrocytes and plasma. Erythrocytes were washed three times with 150 mmol/l NaCl solution. After next centrifugation (4°C, 900 g for 5 min), erythrocytes were hemolyzed by adding the three volumes of distilled water (4°C). Aliquoted plasma and hemolyzed erythrocytes were stored at -20°C until analyses. Concentration of lipid peroxides (LP) and total antioxidant capacity were determined in plasma. Activities of catalase (CAT), glutathione peroxidase (GPx), Cu/Zn superoxide dismutase (SOD), and concentration of hemoglobin (Hb) (Drabkin and Austin, 1932) were determined in hemolysate of erythrocytes.

Plasma LP level was measured as described previously (el-Saadani et al., 1989) and presented in nmol/ml of plasma. Total antioxidant capacity of plasma was determined according to Re et al. (1999) as the trolox equivalent antioxidant capacity (TEAC) and expressed as mmol of trolox/l. Activities of SOD and GPx were determined using a commercially available kits, Fluka, (Germany) and Enzo Life Sciences (United States), respectively, following the instructions of the manufacturers. SOD activity was expressed in units (U) per mg Hb. GPx activity was expressed in μkat/g Hb. Activity of CAT was determined by the modified method by Bergmeyer (1987) and its activity was expressed in μkat/g Hb.

### Vascular Reactivity Measurements

Vascular reactivity of the femoral artery segments (1.0 – 1.5 mm long) was determined using wire myograph (Dual Wire Myograph System 410A, DMT A/S, Aarhus, Denmark). Normalization, including calculations for normalized inner diameter, was performed as described by Puzserova et al. (2013b). The experimental protocol consisted of the following steps: (a) Modified physiological salt solution (PSS, 118.99 mmol/l NaCl, 4.69 mmol/l KCl, 25 mmol/l NaHCO_3_, 1.17 mmol/l MgSO_4_.7H_2_O, 1.18 mmol/l KH_2_PO_4_, 2.5 mmol/l CaCl_2_.2H_2_O, 0.03 mmol/l Na_2_EDTA, 5.5 mmol/l glucose) in the myograph chamber was changed to depolarizing solution KPSS (i.e., PSS in which NaCl was replaced with KCl with concentration of K^+^ 125 mmol/l) for 2 min and contraction was measured. (b) After confirming a sufficient contractile response to KPSS 10 μmol/l of noradrenaline (NA) was added and the phasic and tonic contractions were determined. (c) The segment was pre-constricted with 1 μmol/l 5-hydroxytryptamine (serotonin). Endothelium-dependent relaxation was determined by increasing concentrations of acetylcholine (ACh, from 0.001 to 10 μmol/l), added cumulatively. (d) The same procedure was repeated after pre-incubation (25-min) with 300 μmol/l N^G^-nitro-L-arginine methyl ester (L-NAME, non-specific NOS inhibitor). (e) Endothelium-independent relaxation was determined in 5-hydroxytryptamine pre-constricted (1 μmol/l) arteries by cumulative adding of the NO donor sodium nitroprusside (SNP, 0.001 – 10 μmol/l). (f) Finally, PSS was changed again to KPSS to induce maximal contraction and was left to achieve a plateau. The artery was washed out four times with PSS and stabilized for 20 min after each step and 30 min before creating a SNP-induced concentration response curve. Vasorelaxation was expressed as the percentage of the initial steady-state contraction induced by 5-hydroxytryptamine. Endothelium-dependent relaxations were also determined as the area under the curve (AUC, in arbitrary units) calculated under individual concentration-response relaxation curves. NO-mediated relaxation was determined by measuring the portion of ACh-induced relaxation that was inhibited by L-NAME. Vasoconstrictions were determined as the maximal active wall tension (μN/mm).

Concentrations of all substances were expressed as final concentrations in the myograph chamber. All chemicals used in this study were purchased from Merck Chemicals (Germany) and Sigma-Aldrich (Germany), except for noradrenaline hydrogenotartras (Zentiva, Czechia).

### Statistical Analysis

Heart rate and blood pressure data were analyzed by three-way ANOVA (phenotype × group × day). Relaxation curves were analyzed by three-way ANOVA (phenotype × group × concentration of ACh). All other data were analyzed by two-way ANOVA (phenotype × group). All analysis were followed by Bonferroni’s *post hoc* tests. The significance level of all tests was set at 5% (α = 0.05). Correlation between variables was determined using Pearson’s correlation coefficient (r). All results are presented as the mean ± SEM. Data were analyzed with Statistica^®^ 7.0 (Stat Soft, Inc., United States) and GraphPad Prism 5.0 (GraphPad Software, Inc., United States).

## Results

### Basic Parameters

Blood pressure of 5-week-old WKY and BHR rats differed significantly (104 ± 3 mmHg vs. 127 ± 3 mmHg, *p* < 0.01). There were significant phenotype- and stress-dependent differences in BP and BW of rats, considering the entire duration of the experiment (i.e., fifth to ninth week of rat life). BHR had higher BP (137 ± 2 vs. 111 ± 2 mmHg, *p* < 0.0001, main effect of phenotype) and BW than WKY (180 ± 3 vs. 171 ± 4 g, *p* < 0.05, main effect of phenotype). Stress reduced BW (154 ± 4 vs. 144 ± 3 g, *p* < 0.05, the main effect of stress at the end of stress period).

**FIGURE 1:**
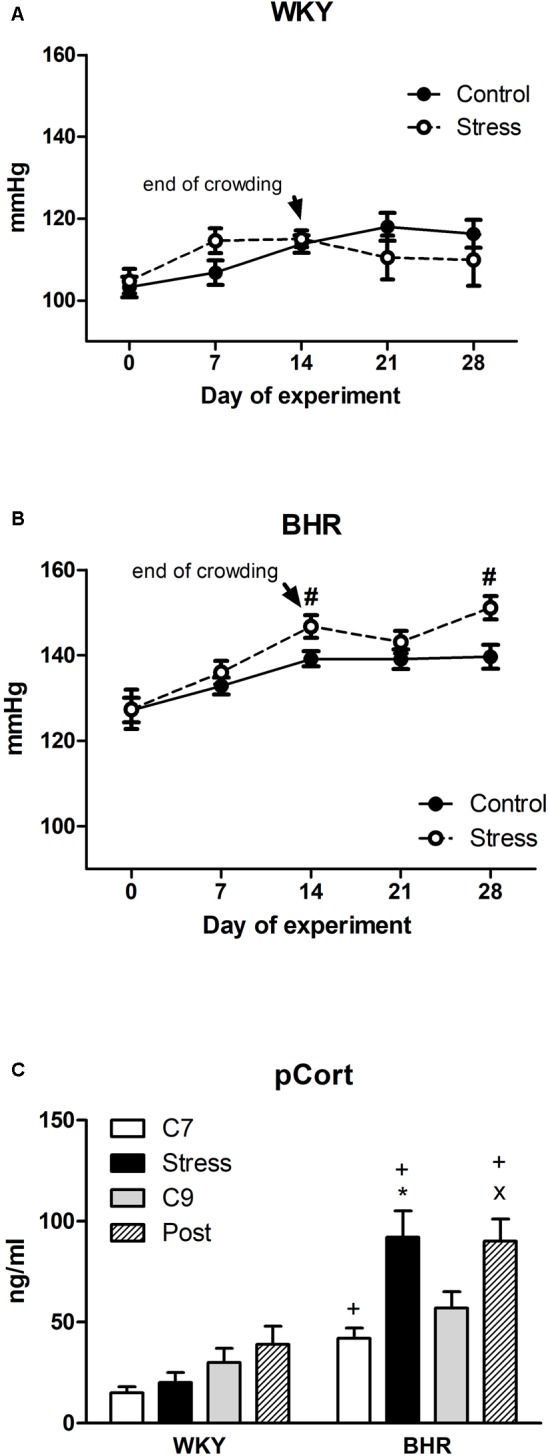
Blood pressure **(A,B)** and plasma corticosterone (pCort, **C**) of normotensive Wistar-Kyoto (WKY) and borderline hypertensive (BHR) rats. Controls (C7 and C9) were kept in control conditions throughout the course of experiment. Stressed rats were exposed to crowding for 2 weeks (days 1–14). After cessation of stress, one group of rats (post-stressed rats, Post) was returned to control conditions for the next 2 weeks. The results are mean ± SEM, *n* = 8–10/group for blood pressure, *n* = 6–8/group for pCort. Symbols: ^#^*p* < 0.05 vs. controls of the same phenotype, ^∗^*p* < 0.05 vs. 7-week controls (C7) of the same phenotype, ^x^*p* < 0.05 vs. 9-week controls (C9) of the same phenotype, ^+^*p* < 0.05 vs. the same WKY group.

Interaction analysis (phenotype x stress x time of exposure) revealed that crowding increased BP significantly in BHR vs. control (*p* < 0.05) but not in WKY on day 14. BP of BHR also remained elevated vs. control (*p* < 0.05) 2 weeks after the cessation of crowding—an effect not seen in WKY (**Figures 1A,B**). The HR of 5-week WKY and BHR rats was 456 ± 10 and 454 ± 8 bpm, respectively, and no significant changes were observed during experiments in either phenotype (data not shown).

Seven-week control BHR had significantly higher pCort levels than age-matched WKY (**Figure 1C**). Stress significantly elevated pCort only in BHR vs. control as well as vs. stressed WKY. High levels of pCort persisted in BHR 2 weeks post stress (**Figure 1C**).

There were no phenotype or stress-related differences in the relative weights of the LHV calculated as the LHV weight-to-BW ratio (**Table 1**). Relative weights of the AGs, calculated as the AGs weight-to-BW ratio, were significantly higher in 7-week control BHR vs. WKY. Relative weights of the AGs were unaffected by stress (**Table 1**).

**Table 1:** Biometric and vascular parameters of the femoral artery in normotensive Wistar-Kyoto (WKY) and borderline hypertensive rats (BHR).

	WKY				BHR			
	C7	Stress	C9	Post	C7	Stress	C9	Post
	*n* = 6–8	*n* = 8–10	*n* = 6–8	*n* = 8–10	*n* = 6–8	*n* = 8–10	*n* = 6–8	*n* = 8–10
ND (μm)	649 ± 17	637 ± 16	681 ± 16	691 ± 10^#^	677 ± 19	659 ± 15	690 ± 11	708 ± 11^#^
E_max ACh_ (%)	87 ± 2	90 ± 2	84 ± 2	84 ± 4	81 ± 3	80 ± 4	83 ± 2	83 ± 3
E_max SNP_ (%)	95 ± 1	97 ± 1	97 ± 1	97 ± 1	97 ± 1	99 ± 1	97 ± 1	96 ± 2
NA_p_ (μN/mm)	518 ± 78	621 ± 48	872 ± 86*	691 ± 174	1253 ± 324^+^	915 ± 148^+^	965 ± 252	643 ± 91
NA_t_ (μN/mm)	71 ± 21	63 ± 11	422 ± 114*	309 ± 105^#^	1429 ± 639^+^	771 ± 269^+^	861 ± 330	1165 ± 549
LHV/BW (mg/g)	1.85 ± 0.05	1.95 ± 0.18	1.56 ± 0.04*	1.63 ± 0.07^#^	1.82 ± 0.07	1.98 ± 0.06	1.65 ± 0.09	1.81 ± 0.03
AG/BW (mg/100 g)	17.1 ± 0.7	16.1 ± 0.3	13.5 ± 0.3*	13.8 ± 3^#^	19.5 ± 0.7^+^	19.3 ± 0.4^+^	16.4 ± 3*	15.7 ± 0.8^#^

*C7, 7-week controls; C9, 9-week controls; Post, Post-stress; E_*max ACh*_, maximal relaxation to acetylcholine based on individual concentration-response curves; E_*max SNP*_, maximal relaxation to sodium nitroprusside based on individual concentration-response curves; NA_*p*_, maximal phasic response to noradrenaline; NA_*t*_, maximal tonic response to noradrenaline; ND, normalized internal diameter of the femoral artery at 13.3 kPa; SNP, sodium nitroprusside; AG/BW, adrenal glands weight-to-body weight ratio; LHV/BW, left heart ventricle weight-to-body weight ratio. The results are mean ± SEM. ^∗^*p* < 0.05 vs. seven-week controls (C7) of the same phenotype, ^#^*p* < 0.05 vs. stress of the same phenotype, ^+^*p* < 0.05 vs. WKY of the same group.*

### NO Synthase Activity

**FIGURE 2:**
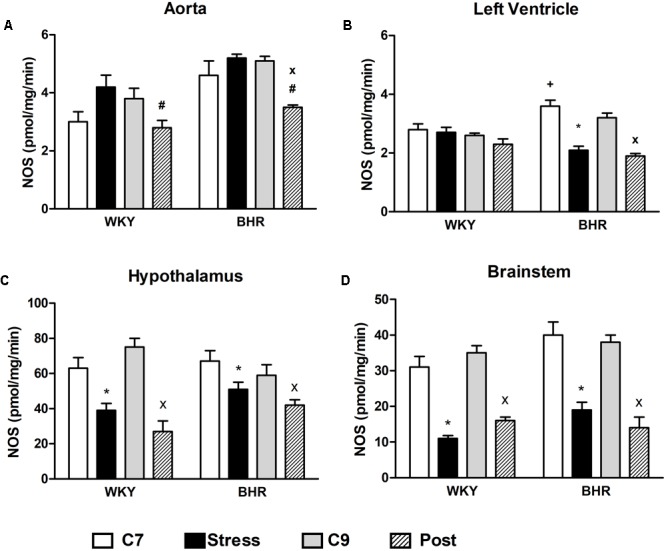
Nitric oxide synthase (NOS) activity in the aorta **(A)**, left heart ventricle **(B)**, hypothalamus **(C)** and brainstem **(D)** of normotensive Wistar-Kyoto (WKY) and borderline hypertensive (BHR) rats in 7-week controls (C7, white bar), stress (black bar), 9-week controls (C9, gray bar) and post-stress (Post, strip bar). In the aorta, the main effect of stress vs. age-matched controls (C7) was significant (see Results section for details). The results are mean ± SEM, *n* = 6–8/group. Symbols: ^∗^*p* < 0.05 vs. 7-week controls (C7) of the same phenotype, ^#^*p* < 0.05 vs. stress of the same phenotype, ^x^*p* < 0.05 vs. 9-week controls (C9) of the same phenotype, ^+^*p* < 0.05 vs. the same WKY group.

In the aorta, NOS activity tended to be increased in stressed rats of both phenotypes, resulting in a significant main effect of stress vs. age-matched controls (4.85 ± 0.33 vs. 3.41 ± 0.35 pmol/mg/min, p < 0.05, main effect of stress, **Figure 2A**). In the LHV stress reduced NO synthase activity only in BHR (**Figure 2B**). In the hypothalamus and brainstem (**Figures 2C,D**) stress reduced NOS activities in both phenotypes. At the end of post-stress recovery, significantly reduced NOS activities were detected in the all tissues investigated in BHR vs. age-matched controls (**Figures 2A–D**). In WKY, a significant decrease of NOS activity in the hypothalamus and brainstem was observed in the post-stress group compared to age-matched controls (**Figures 2C,D**).

### Oxidative Status

**FIGURE 3:**
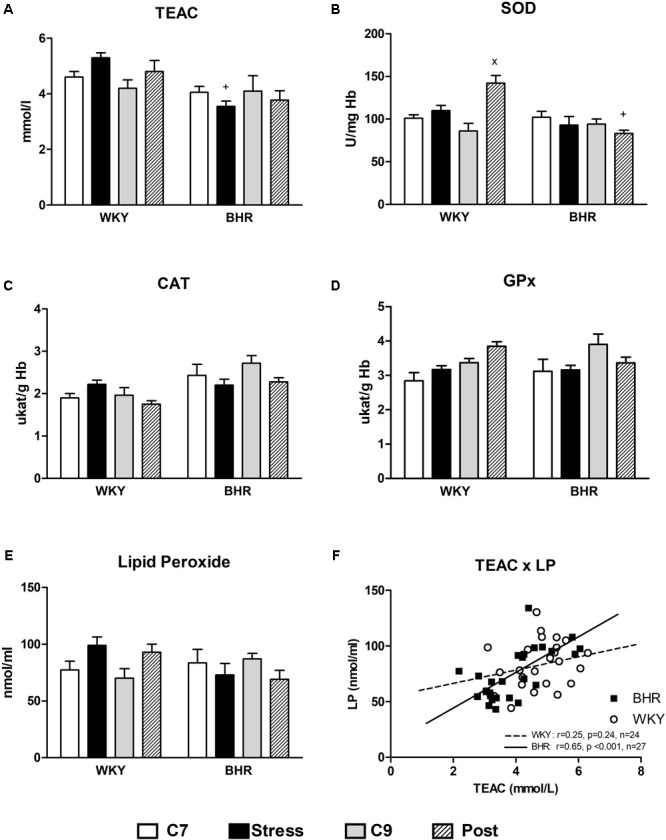
Parameters of oxidative status **(A–E)** in the blood of normotensive Wistar-Kyoto (WKY) and borderline hypertensive (BHR) rats in 7-week controls (C7, white bar), stress (black bar), 9-week controls (C9, gray bar) and post-stress (Post, strip bar). **(F)** Shows correlations between TEAC and LP in WKY and BHR rats. CAT, catalase; GPx, glutathione peroxidase; Hb, hemoglobin; LP, lipid peroxides; SOD, Cu/Zn- superoxide dismutase; TEAC, trolox equivalent antioxidant capacity of plasma. The results are mean ± SEM, *n* = 6–7/group. Symbols: ^x^*p* < 0.05 vs. nine-week controls (C9) of the same phenotype, ^+^*p* < 0.05 vs. the same WKY group.

No differences in SOD, CAT and GPx activities in erythrocytes as well as in plasma TEAC and LP were found in 7- and 9-week-old control WKY and BHR (**Figures 3A–E**). Stress reduced TEAC in BHR compared to stressed WKY (**Figure 3A**). SOD activity was significantly elevated in post-stressed WKY vs. age-matched controls and post-stressed BHR (**Figure 3B**). There was a significant positive correlation between TEAC and LP in BHR (*r* = 0.65, *p* < 0.001, *n* = 27) which was not present in WKY rats (*r* = 0.25, *p* = 0.24, *n* = 24, **Figure 3F**).

### Vascular Reactivity

Normalized internal diameter of the femoral artery was similar in age-matched control WKY and BHR. Stress exposure had no effect on this parameter (**Table 1**).

Both endothelium-dependent ACh-induced and endothelium-independent SNP-induced maximal relaxations were similar in all groups investigated (**Table 1**). Five-hydroxytryptamine-induced vasoconstriction was similar among groups (results not shown). NA-induced responses of the femoral artery were biphasic: a transient phasic contraction, which returned nearly to baseline, was followed by sustained tonic contraction. Both NA-induced phasic and tonic responses were elevated in control BHR vs. WKY (*p* < 0.03, main effect of phenotype in all control rats), but this effect was more pronounced in 7-week control rats (**Table 1**). No effects of stress and post-stress recovery on NA-induced vasoconstriction were observed compared to age-matched controls. Similar results for NA contractions were obtained in relative values calculated as percentage of maximal response induced by KPSS (results not shown).

**FIGURE 4:**
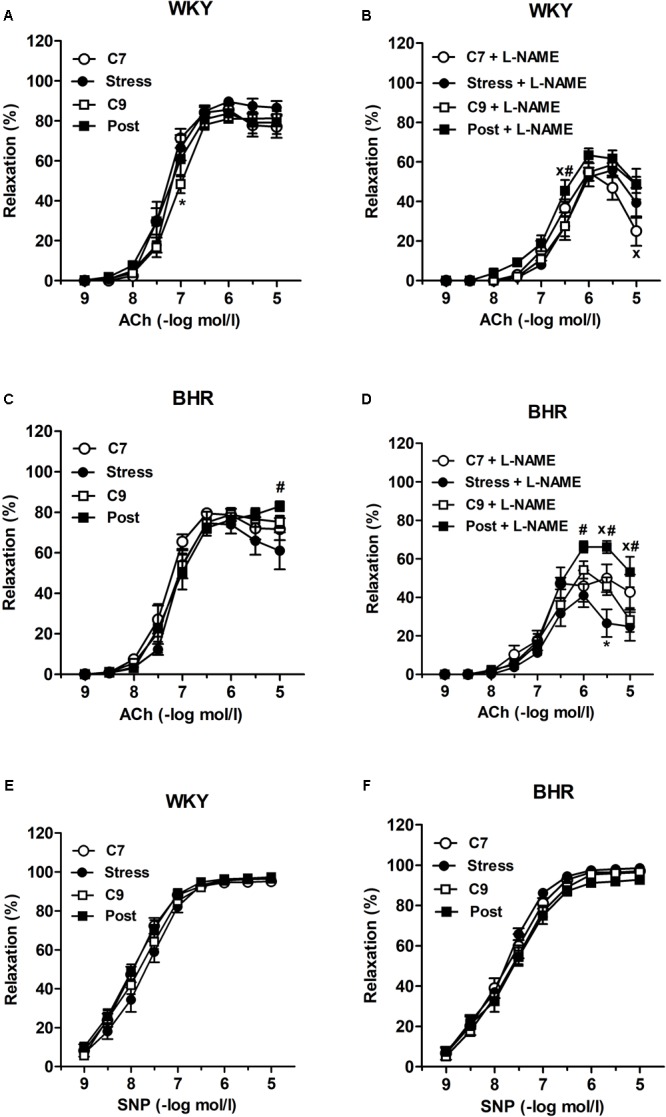
Vascular responses to acetylcholine **(A–D)** and sodium nitroprusside **(E,F)** in the isolated femoral arteries of normotensive Wistar-Kyoto (WKY) and borderline hypertensive (BHR) rats before **(A,C)** and after **(B,D)** incubation with nitric oxide synthase inhibitor N^G^-nitro-L-arginine methyl ester (L-NAME, 300 μmol/l). ACh, acetylcholine; C7, seven-week controls; C9, nine-week controls; Post, post-stress; SNP, sodium nitroprusside. The results are mean ± SEM, *n* = 6–10/group. Symbols: ^∗^*p* < 0.05 vs. seven-week controls (C7) of the same phenotype,^#^*p* < 0.05 vs. stress of the same phenotype, ^x^*p* < 0.05 vs. nine-week controls (C9) of the same phenotype.

**FIGURE 5:**
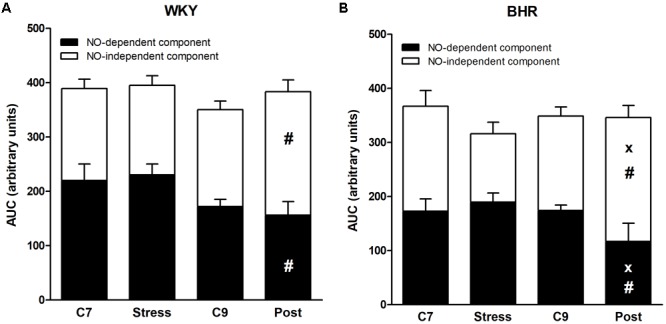
Acetylcholine-induced relaxation of the femoral artery of normotensive Wistar-Kyoto (WKY, **A**) and borderline hypertensive (BHR, **B**) rats. AUC, area under the curve; C7, seven-week controls; C9, nine-week controls; NO, nitric oxide; Post, post-stress. The results are mean ± SEM, *n* = 6–10/group. Symbols: ^#^*p* < 0.05 vs. stress of the same phenotype, ^x^*p* < 0.05 vs. nine-week controls (C9) of the same phenotype.

There were no considerable differences in ACh and SNP-induced concentration-response curves and maximal relaxations among the groups (**Table 1** and **Figures 4A,C,E,F**). Application of L-NAME partially inhibited the ACh-induced relaxation in all groups investigated, as illustrated in **Figures 4B,D**. However, L-NAME attenuated ACh-induced relaxation less markedly in post-stressed BHR than in age-matched controls (**Figure 4D**). Furthermore, the reduced NO-dependent component of ACh-induced relaxation was observed in both post-stressed WKY and BHR vs. the respective stress group, as well as in post-stressed BHR vs. the age-matched control BHR group (**Figures 5A,B**). Conversely, the NO-independent component of vasorelaxation induced by ACh was elevated in the BHR post-stress group vs. the respective age-matched control BHR group, which fully compensated for the decrease of NO-dependent relaxation of the femoral arteries (**Figures 4D, 5B**).

## Discussion

This study showed that 5-week-old male BHR rats—offspring of SHR dams and WKY sires—had significantly elevated BP compared to WKY (i.e., offspring of two normotensive parents). However, there were no differences in activities of enzymes involved in antioxidant defense system, antioxidant capacity of plasma, plasma lipid peroxidation, or endothelium-dependent relaxations of the femoral arteries. Also, there were no signs of reduced NO production in the aorta, LHV, hypothalamus or brainstem in either 7- or 9-week-old control BHR compared to normotensive controls, excluding these pathologies as a cause of BP increase in BHR rats. However, pCort concentrations were elevated in control BHR vs. WKY under restful conditions. In addition, BP and pCort were significantly increased by crowding only in BHR, remaining elevated 2 weeks post-stress, thereby suggesting that neurogenic mechanisms associated with HPA axis perturbance play a role in the initiation of BP rising.

Regarding endothelial function, similar to our above findings, no changes in overall endothelium-dependent relaxation of small mesenteric arteries were found in 15–17-week-old BHR (Fuchs et al., 1998). However, in adult 22-week-old BHR males, in which systolic BP reached ∼140 mmHg, both ED and LHV hypertrophy were determined (Puzserova et al., 2013a), suggesting that ED and LHV hypertrophy in adulthood are the consequences of pressure overload in this genetic model of borderline hypertension (Bernatova, 2014). Furthermore, no signs of reduced NO production in the aorta were observed in this study in the control BHR, in association with unchanged NO-dependent vasorelaxation of the femoral arteries, demonstrating that vascular NO deficiency is not primarily involved in the initiation of BP increases in young BHR. Similarly, no changes in antioxidant defense system enzymes activities or plasma LP concentrations were detected in 7-week-old BHR suggesting that oxidative stress is not involved in the initiation of BP elevation in this strain. However, there are data that suggest an important role of glucocorticoids in the control of BP, as they may increase the pressor response to noradrenaline, angiotensin II and other vasoconstrictors (Puzserova and Bernatova, 2016). In our study, relative weights of the AGs, pCort levels and NA-induced constrictions were higher in 7-week-old control BHR than in WKY rats, which may be involved in the initiation of BP increases.

Exposure to stress produced by crowding was used in our study to investigate whether stress can induce the development of hypertension. We investigated possible involvement of stress in the development of both NO deficiency in the cardiovascular system and ED in young male BHR. Recent studies showed elevated NO production and NO-dependent relaxations in adult normotensive rats exposed to stress (Puzserova et al., 2013b; Bouzinova et al., 2014) in various types of arteries. In this study, endothelial function and NO-dependent relaxations were similar in young rats of both strains exposed to stress, possibly because of the shorter duration of the stress. Aortic NO production in stressed rats vs. controls was elevated in both strains when calculated as the main effect of stress. In contrast to the findings in the aorta, stress reduced cardiac NO productions in young BHR males in this study. We have shown previously that long-term cardiac NO deficiency results in structural changes such as development of fibrosis and LHV hypertrophy (Babal et al., 1997). Altered cardiac structure, in association with functional changes, can contribute to the maintenance of high blood pressure, even after cessation of the stressor. Indeed, elevated BP and reduced NO production in the LHV were present in BHR 2 weeks post-stress, which was in contrast to WKY rats. In addition, reduced aortic NO production was observed in the aortae 2 weeks post-stress, which was associated with a reduced NO-dependent component of relaxations of the femoral arteries, suggesting a delayed decrease of vascular NO bioavailability in the conduit arteries of BHR. This may result from the long-term glucocorticoid overload, which can produce alterations in the vascular function. For example, exposure to dexamethasone, which is a synthetic glucocorticoid, reduced eNOS expression in the endothelial cell cultures (Wallerath et al., 1999). Simmons et al. (1996) found that dexamethasone reduced iNOS-mediated NO production in the endothelial cells exposed to cytokines, which was associated with reduction in the tetrahydrobiopterin (NOS cofactor) synthesis. In addition, in the isolated mesenteric arteries, dexamethasone reduced nNOS-mediated NO release in the arteries from spontaneously hypertensive but not normotensive rats (Aras-López et al., 2009). Recently, Lee et al. (2015) has revealed that chronic stress induced by restraint resulted in the reduction in the pial artery dilatation that was associated with a significant reduction of nNOS protein expression, without significant changes in eNOS and iNOS expressions. These studies suggest diverse effects of chronic glucocorticoid overload on vascular NO production, which may differ in normotensive and (pre)hypertensive subjects. As this study was not primarily focused on the role of individual NOSs in stress-induced responses, the contributions of individual NOSs in crowding stress-induced NO-dependent vascular changes in the conduit arteries remains to be elucidated.

However, in our study, the decreases in NO-dependent component of vasorelaxation were fully compensated by NO-independent mechanisms, presumably by elevated prostacyclin- and/or endothelium-derived hyperpolarizing factor-mediated relaxations (Bouzinova et al., 2014). We showed that the impairment of overall endothelial function does not contribute to the maintenance of high BP in young, post-stress BHR, despite altered mechanism(s) mediating endothelium-dependent relaxations. However, it is worth noting that reduced NO bioavailability may be implicated in various endocrine/metabolic disorders, independently of BP. Thus, this mechanism may provide a link between the delayed effects of chronic stress and other stress-induced disorders such as atherosclerosis or insulin resistance (Kawashima and Yokoyama, 2004; Kim et al., 2006).

Consistent with the unchanged vasorelaxations, no alterations in oxidative status were observed in stressed rats in this study. However, SOD activities in post-stressed BHR were significantly reduced compared to those in post-stressed WKY. This might be involved in slow progress of oxidative damage to plasma LP in BHR, consistent with the positive correlation between total antioxidant capacity of plasma and LP. A similar correlation was observed previously in rats with various genetic predispositions to hypertension (Horvathova et al., 2016), and may play an important role in hypertension maintenance in later life. In addition, the lack of oxidative damage to lipids in blood does not exclude local tissue oxidative stress. Specifically, oxidative stress in the brain can contribute to hypertension development via increased sympathoexcitation (Kishi and Hirooka, 2012).

We found that crowding led to significant increases in pCort and an acceleration of BP rise only in BHR. Similarly, higher pressor responses to various stressors have been shown previously in adult BHR males compared with WKY (Fuchs et al., 1998; Bechtold et al., 2009; Sarenac et al., 2011). Because crowding stress failed to induce oxidative stress and ED, the differences in crowding-induced BP increases might be related to the central effects of corticosterone. Bechtold et al. (2009) found that endogenous corticosterone acts via hindbrain glucocorticoid receptors to increase the blood pressure responses to stress in adult BHR males, in contrast to WKY.

In this study, crowding stress elevated BP and pCort while reducing NO production in the hypothalamus and brainstem in BHR. NO is an important mediator in regulation of the HPA axis under both rest and stress, that is involved in HPA axis attenuation and activation, respectively (for review see Puzserova and Bernatova, 2016).

Regarding delayed effect of stress, in our study all BP, pCort and NO production in the selected brain areas failed to recover 2 weeks post-stress. Persistent increases of pCort after stress may result from epigenetic mechanisms such as DNA methylation of glucocorticoid receptors (Turecki and Meaney, 2016), which may impede the HPA axis negative feedback regulation. Subsequently, chronic high glucocorticoid levels may decrease NO bioavailability in various brain areas. It has been shown that high levels of glucocorticoids led to alterations in neuronal NO release in the central nervous system, which is an important mechanism in development of hypertension (Goodwin and Geller, 2012).

Thus, the interaction of stress-induced NO insufficiency in the vascular and central nervous system with accentuated noradrenergic function and sympathetic nervous system (SNS) excitation may provoke BP increases in BHR, similar to those in unstressed SHR. In SHR, altered noradrenergic function and increased SNS tone can be considered to be mechanisms underlying hypertension (Hojna et al., 2007; Shinohara et al., 2012; Sterley et al., 2016).

## Conclusion

This study yields several important results. First, we found that generalized oxidative stress, vascular NO deficiency and ED are not causally involved in the initiation of BP increases in BHR, as these pathologies were not present in 7-week or 9-week control BHR despite significantly elevated BP vs. young normotensive rats.

Second, we observed that exposure to stressful environments reduced NO production in the hypothalamus and brainstem of WKY without increasing BP. In contrast, the same environments in BHR resulted in persistent increases in pCort, reductions in brain and cardiac NO production, and delayed alterations in endothelial function that worked together to accelerate the increase of BP in BHR. Thus, relatively short-term, mild social stress, experienced in early life, can create persistent impairment in HPA axis regulation followed by accelerations of BP increase in the offspring of hypertensive mothers and normotensive fathers.

## Author Contributions

IB designed the research studies, conducted selected experiments, performed statistical analysis, and wrote the manuscript. AP and IZ participated in statistical analysis and writing of the manuscript. AP, PB, NS, MH, and ZK carried out the studies and analyzed the data. IB had primary responsibility for the final content. All authors read and approved the final manuscript.

## Conflict of Interest Statement

The authors declare that the research was conducted in the absence of any commercial or financial relationships that could be construed as a potential conflict of interest.
